# Symptoms of common mental disorders and suicidality among sexually diverse men who have sex with men in Ghana

**DOI:** 10.1371/journal.pmen.0000331

**Published:** 2025-07-03

**Authors:** Melissa A. Stockton, Sangchoon Jeon, Samuel Akyirem, Edem Zigah, Nii Dromo Wallace-Atiapah, Gamji Rabiu Abu-Ba’are, Richard Panix Amo-Oto, Irene Ofori, Michael Adu, Kwasi Torpey, Laura Nyblade, LaRon E. Nelson

**Affiliations:** 1 Gillings School of Global Public Health, University of North Carolina at Chapel Hill, Chapel Hill, North Carolina, United States of America; 2 School of Nursing, Yale University, New Haven, Connecticut, United States of America; 3 School of Nursing, University of Rochester, Rochester, New York, United States of America; 4 Priorities on Rights & Sexual Health, Accra, Ghana; 5 Youth Alliance for Health & Rights, Kumasi, Ghana; 6 Department of Population, Family & Reproductive Health, School of Public Health, University of Ghana, Legon-Accra, Ghana; 7 RTI International, Washington, Columbia, United States of America; 8 Yale Institute for Global Health, School of Public Health, New Haven, Connecticut, United States of America; PLOS: Public Library of Science, UNITED KINGDOM OF GREAT BRITAIN AND NORTHERN IRELAND

## Abstract

In Ghana, as in many countries globally, sexually diverse, gay and bisexual men who have sex with men (MSM) face a syndemic of HIV and mental illness. This global syndemic of HIV and poor mental health among MSM is driven by social and structural processes, including stigma, human rights abuses, and interpersonal violence that exacerbate marginalization, poverty, and illness and hamper access to health services. Many countries like Ghana lack prevalence data documenting the burden of mental health challenges among MSM. This cross-sectional study followed a waitlist-controlled randomized control trial (RCT) of a multilevel intersectional (HIV, sexual, and gender nonconformity) stigma-reduction intervention for healthcare facility staff and MSM to improve HIV testing in Accra and Kumasi, Ghana. Participants were screened for depression, anxiety and PTSD with the Patient Health Questionaire-9, General Anxiety Disorder-7, and Primary Care Post Traumatic Stress Disorder Screen for DSM-5 (PC-PTSD-5). Univariate analyses were conducted to assess the prevalence of symptoms of depression, suicidality, anxiety, and PTSD by HIV status. 186 participants completed the mental health survey. Among the sample, high prevalence of mental health concerns was reported: 52.1% mild to severe depression; 35.0% suicidality; 50.5% mild to severe anxiety; and 29.8% PTSD. The prevalence was higher among those who were living with HIV compared to HIV negative: moderate to severe depression 25.7 vs. 20.5%; suicidality 45.7% vs. 36.1%; moderate to severe anxiety 9.5% vs. 14.3%; and PTSD 50.0% vs. 30.8%. Investigation of risk and protective factors and investment in mental healthcare for MSM is needed.

## Introduction

Sexually diverse, gay and bisexual men who have sex with men (MSM) face co-occurring and synergistic epidemics of HIV and mental health problems across the globe [1]. This global syndemic of HIV and poor mental health among MSM is driven by their overexposure to a host of inequitable multi-level social and structural processes, namely stigma, discrimination, human rights abuses, and interpersonal violence that cumulatively exacerbate marginalization, poverty, and illness and restrict access to necessary health services [2–4]. A small, growing body of research out of Africa is beginning to document these interlocking systems and their impact on HIV and mental health [5–8]; however, many African countries, including Ghana, lack even prevalence data. In Ghana, 18% of MSM are estimated to be living with HIV, with an estimated HIV prevalence as high as 42% among MSM in Greater Accra [4]. Further, Ghanaian MSM face severe stigma and discrimination, that remains codified in law, further driving inequity in health, well-being, socio-emotional development, and economic progress [9–12]. Yet, protecting the mental health of this vulnerable population has received little attention. As such, this short report aims to document the prevalence of symptoms for depression, suicidality, anxiety, experienced trauma, and post-traumatic stress disorder (PTSD) among a sample MSM in Ghana.

## Materials and methods

This study followed a waitlist-controlled randomized control trial (RCT) of a multilevel intersectional (HIV, sexual, and gender nonconformity) stigma-reduction intervention for healthcare facility staff and MSM to improve HIV testing Ghana. [12] While the RCT focused specifically on stigma and HIV outcomes, the formative work as well as engagement in the intervention revealed that mental health was a prime concern for MSM. As such, we were able to leverage the completion of the RCT to conduct this small, cross-sectional survey to document the prevalence of mental health concerns. In this short report, we briefly describe the intervention that the mental health survey participants received in the study population section. We then present the methods for the cross-sectional mental health survey.

### Purpose

This study aimed to measure symptoms of depression, suicidality, anxiety, and PTSD among MSM in Ghana. We hypothesized that there would be a high prevalence of these mental health concerns among this population.

### Study population

Participants included in this cross-sectional survey completed two stigma-reduction interventions: interpersonal-level *Nyansapo,* a Ghanian adaptation of the Many Men, Many voices (3MV) intervention [12,13], and individual-level *HIV Empathy Education & Empowerment (HIVE*^*3*^*)* [14]. *Nyansapo* consisted of peer, group sessions of brainstorming, role playing, small group and paired activities, and action planning on the following topics: 1) Ghanaian MSM and dual identity; 2) HIV/STD prevention for Ghanaian MSM; 3) HIV/STD risk assessment and prevention options; 4) Intentions to act and capacity for change; 5) Relationship issues: Partner selection, communication and negotiation of roles; 6) Social support and problem solving to maintain change; 7) Building bridges and community [12]. *HIVE*^*3*^ consisted of individual, one-on-one phone based text or voice contacts, in which MSM study participants reached out to trained peer mentored reached out to MSM participants for informal discussions on affirming identities, information regarding HIV prevention and testing and overring emotional support [12]. The intervention group received the intervention between August 2021 and January 2022 and the control group received the intervention between August 2022 and December 2022.

Participants eligible for the mental survey included adult MSM (ages 18 or older) who participated in the RCT [12]. All participants lived in either the Kumasi or Accra metropolitan areas, were assigned male sex at birth, and reported sexual activity with another man at least once within the previous six months of enrollment into the trial. All participants had completed all intervention activities at the time of the survey.

### Mental health and sociodemographic measures

#### Patient Health Questionaire-9 (PHQ-9).

The PHQ-9 includes nine questions that assesses the presence and frequency of the nine Diagnostic and Statistical Manual of Mental Disorders (DSM) symptoms of major depression. A total score of 5–9, 10–14, and ≥15 are considered indicative of mild, moderate, and severe depression, respectively [15]. Any endorsement of question 9, which probes on thoughts of being better off dead or of hurting oneself, is indicative of suicidality. In this sample, the PHQ-9 demonstrated good reliability (Cronbach’s α = 0.82).

#### Generalized Anxiety Questionnaire-7(GAD-7).

The GAD-7 includes seven questions that assesses the presence and frequency of the DSM symptoms of general anxiety disorder [16]. A total GAD-7 score of 5–9, 10–14, and ≥15 are considered indicative of mild, moderate, and severe anxiety, respectively. In this sample, the GAD-7 demonstrated good reliability (Cronbach’s α = 0.82).

#### Primary Care Post Traumatic Stress Disorder screen for DSM-5 (PC-PTSD-5).

The PC-PTSD-5 includes six questions; an opening question that asks about prio traumatic experiences followed by a five questions that assess the core DSM-5 symptoms of PTSD asked only of those who report trauma [17]. Total scores of ≥ 4 are considered indicative of PTSD. In this sample, the PC-PTSD-5 demonstrated good reliability (Cronbach’s α = 0.88).

#### Sociodemographic data.

Participants provided self-reported sociodemographic information (age, education, residency, gender identity, sexual orientation), sexual behavior, and HIV status on the baseline survey at enrollment into the RCT.

### Data collection procedures

Data collection took place between 5 November 2022 and 3 January 2023, after all participants had received the intervention. Research assistants from the MSM-serving organizations that had managed data collection during the RCT reached out to the intervention participants by phone to invite them to participate in the mental health survey. In Kumasi, research assistants organized set times at different public locations for data collection, whereas in Accra research assistants met with participants individually at their homes or at the community partner’s office. Participants self-administered the survey in English on tablets using the password protected CommCare application. Research assistants, fluent in both English and the local languages (e.g., Twi, Ga) were available to clarify any questions participants had about the survey items. Participants’ responses could be linked to their RCT survey data via a unique alphanumeric study identification code. Research assistants were blinded to the participants responses to the surveys. All participants received referral information on where to access mental health counseling services.

### Analysis

Univariate analyses were conducted to assess the prevalence of symptoms of depression, suicidality, anxiety, and PTSD by HIV status.

Missing imputation was performed for PHQ-9, GAD-7, and PC-PTSD-5 scores separately. For participants missing ≤3 PHQ-9 items, ≤ 2 GAD-7 items, ≤ 1 PC-PTSD-5 items, the missing items were imputed using Monte Carlo Markov Chain (MCMC) approach (assuming Missing at Random) based on the item mean and covariance between items.

### Ethics

The Yale University, University of Ghana Noguchi Memorial Institute, Ghana Health Services, and University of Toronto Institutional Review Boards approved this study. All participants provided written informed consent. Study participants received 200 Ghanaian Cedis (19 US dollar equivalent) for their participation.

## Results

### Participant characteristics

186 participants of the parent RCT completed the mental health survey (**Table 1**). The average age was 25.2 years old (range 18–45). Approximately half of participants (n = 94) resided in Accra, while the remaining 48.2% (n = 91) resided in Kumasi. A large proportion (86%; n = 160) had completed at least senior secondary education. With respect to gender identity, sexual behavior, and sexual orientation: 81.6% (n = 151) identified as a man, 2.2% (n = 4) identified as a woman, 15.7% (n = 29) identified as both man and woman and 0.5% (n = 1) identified as neither man or woman; 49.7% (n = 89) reported sex with only men; 50.5% (n = 94) identified has gay, 40.5% (n = 75) identified as bisexual, 4.3% (n = 8) identified as straight, 1.1% (n = 2) were not sure about their sexual orientation, and 3.8% (n = 7) did not want to answer. With respect to HIV status, 18.8% (n = 35) reported they were living with HIV, 45.2% (n = 84) reported they were HIV negative, 26.3% (n = 49) reported they did know, and 9.7% (n = 18) did not want to answer.

**Table 1:** Participant characteristics (N = 186).

	Mean (SD), Median [Min, Max] or N (%)
Age	25.2 (4.5), 24.0 [18, 45]
**Education**	
No formal education	1 (0.5%)
Primary Education	4 (2.2%)
Middle/Junior High School	21 (11.3%)
Senior Secondary	99 (53.2%)
Tertiary	61 (32.8%)
**Residence** ^*^	
Accra	94 (50.8%)
Kumasi	91 (48.2%)
**Gender Identity** ^*^	
Man	151 (81.6%)
Woman	4 (2.2%)
Both Man and Woman	29 (15.7%)
Neither	1 (0.5%)
**Sexual Behavior (Who do you have sex with?)** ^**^	
Men only	89 (49.4%)
Both Men/Women	91(50.6%)
**Sexual Orientation**	
Gay	94 (50.5%)
Straight	8 (4.3%)
Bisexual	75 (40.3%)
Not Sure	2 (1.1%)
Don’t want to answer	7 (3.8%)
**HIV Status**	
HIV Positive	35 (18.8%)
HIV Negative	84 (45.2%)
I don’t know	49 (26.3%)
I don’t want to answer	18 (9.7%)

* missing n=1;

** missing n= 5

### Prevalence of symptoms of Common Mental Disorders (CMDs)

Of the 186 participants, 186 contributed PHQ-9 and GAD-7 scores and 168 contributed PC-PTSD-5 scores. With respect to missingness on the PHQ-9, six participants were missing responses to one question and one participant was missing responses to two questions. All PHQ-9 scores for the participants with missing PHQ-9 data were imputed. One participant was missing a response to the ninth question of the PHQ-9. For the GAD-7, 52 participants were missing responses to one question and two participants were missing responses to two questions. All GAD-7 scores for the participants with missing data were imputed. For the PC-PTSD-5, 18 participants were missing responses to all of the PC-PTSD-5 questions and four participants who reported a traumatic event were missing responses to one question. Only PC-PTSD-5 scores for the four participants missing responses to one question were imputed. See S1 Appendix for further details on the assessment of missingness and the impact of imputation.

Among the sample, the prevalence of symptoms of mild to severe depression was 52.1% and the prevalence of symptoms of moderate to severe depression was 20.4% (**Table 2**). Further, 35.1% of participants reported suicidality on the 9th item of the PHQ-9. The prevalence of probable mild to severe and moderate to severe anxiety were 50.5% and 12.3%, respectively. With respect to PTSD, 62.5% of participants reported ever experiencing a traumatic event and the prevalence of symptoms of PTSD was 31.5%. Psychiatric multimorbidity was common; 50.6% reported at least two probable common mental disorders (CMDs).

**Table 2:** Prevalence of symptoms of depression, anxiety and PSTD, by HIV status.

N (%, 95% CIs)	Total	HIV Status		
		Negative	Positive	Don’t Know
		(n = 84)	(n = 35)	(n = 49)
**Depression (n = 186)**				
Mild (PHQ-9 5–9)	59 (31.7, 25-38.4%)	32 (38.1, 27.7-48.5%)	10 (28.6, 13.6-43.5%)	14 (28.6, 15.9-41.2%)
Moderate (PHQ-9 10–14)	27 (14.5, 9.5-19.6%)	12 (14.3, 6.8-21.8%)	7 (20.0, 6.7-33.3%)	6 (12.2, 3.1-21.4%)
Severe (PHQ-9 ≥ 15)	11 (5.9, 2.5-9.3%)	5 (6.0, 0.9-11%)	2 (5.7, -2.0-13.4%)	2 (4.1, -1.5-9.6%)
**Suicidality** (Q9 +) (n = 185)	65 (35.1, 28.3-42%)	30 (35.7, 25.5-46%)	16 (45.7, 29.2-62.2%)	13 (26.5, 14.2-38.9%)
**Anxiety (n = 186)**				
Mild (GAD-7 5–9)	71 (38.2, 31.2-45.2%)	42 (50, 39.3-60.7%)	13 (37.1, 21.1-53.2%)	11 (22.4, 10.8-34.1%)
Moderate (GAD-7 10–14)	20 (10.8, 6.3-15.2%)	6 (7.1, 1.6-12.7%)	5 (14.3, 2.7-25.9%)	7 (14.3, 4.5-24.1%)
Server (GAD-7 ≥ 15)	3 (1.6, -0.2-3.4%)	2 (2.4, -0.9-5.6%)	0	1 (2.0, -1.9-6%)
**PTSD (n = 168)**				
Experienced traumatic event	105 (62.5, 55.2-69.8%)	48 (61.5, 50.7-72.3%)	24 (80, 65.7-94.3%)	21 (50.0, 34.9-65.1%)
PTSD (PC-PTSD-5 ≥ 4)	53 (31.5, 24.5-38.6%)	24 (30.8, 20.5-41%)	15 (50, 32.1-67.9%)	9 (21.4, 9-33.8%)
**Number of CMDs (n = 168)**				
1	19 (11.3, 6.5-16.1%)	9 (11.5, 4.4-18.6%)	5 (16.7, 3.3-30%)	3 (7.1, -0.6-14.9%)
2	54 (32.1, 25.1-39.2%)	27 (34.6, 24.1-45.2%)	8 (26.7, 10.8-42.5%)	12 (28.6, 14.9-42.2%)
3	34 (20.2, 14.2-26.3%)	17 (21.8, 12.6-31%)	9 (30, 13.6-46.4%)	5 (11.9, 2.1-21.7%)

CMD = Common Mental Disorders, inclusive of mild to severe depression, mild to severe anxiety, and PTSD

The prevalence of symptoms of CMDs and suicidality was higher among those who self-reported they were living with HIV compared to HIV negative: moderate to severe depression 25.7 vs 20.5%; suicidality 45.7% vs 36.1%; moderate to severe anxiety 14.3% vs 9.5%; and PTSD 50.0% vs. 30.8%.

## Discussion

This small cross-sectional study documented a high prevalence of symptoms of CMDs among a non-probability sample of MSM in Ghana. In comparison, only around 10% of Ghana’s entire population is estimated to have any mental disorder. [18] Our findings corroborate the limited research from sub-Saharan Africa, and fill a gap in available data from West Africa. For example, in South Africa, MSM similarly reported a high prevalence of depression (44%) and suicidality (56%); in Lesotho 16% of MSM reported depression; in Tanzania, 46.% of MSM reported elevated depressive symptoms; and in Kenya, 53.2% of sexual and gender minority people reported PTSD and 26.1% depression [6,19–21]. These astoundingly high reports of depression, suicidality, anxiety, experienced trauma and PTSD demonstrate a critical unmet need for mental health care. Psychosocial interventions have shown efficacy at improving mental health among MSM [22]. Sexual health programming for MSM presents a clear opportunity for integration of mental health services to address the complex needs of this vulnerable population [1].

Our findings also support research suggesting a higher prevalence of CMDs – particularly depression – among MSM who are living with HIV [23,24]. Many factors may contribute to elevated risk of CMDs among MSM living with HIV including access to MSM-friendly HIV treatment, HIV-related stigma, isolation, and exclusion – even from within the MSM community, social support, opportunistic infections, unemployment, stress and poverty [21,25–28]. However, very limited research unpacking the mechanisms underlying CMDs among MSM living with HIV in sub-Saharan Africa – particularly within West Africa – has been conducted [23]. While our study was unfortunately neither powered nor designed to explore factors driving differences in CMDs by HIV status, further investigation of risk and protective factors for mental health broadly – inclusive of unhealthy substance use – is urgently needed.

### Limitations

The study employed non-diagnostic mental health screening measures to assess prevalence at standard cutoff scores. While the PHQ-9 and GAD-7 have been used in Ghana [29–31], the screeners have yet to be validated against a diagnostic gold-standard in Ghana. While it is possible that imputation of missing data, particularly on the GAD-7, may have biased the prevalence estimates, the expected impact of imputation on total scores is minimal (see S1 Appendix). This analysis includes a unique population of MSM – participants from an intervention trial. The mental health survey participants had recently participated in a stigma-reduction and HIV prevention intervention that targeted intra- and inter-personal intersectional stigma and encouraged peer-support. Given the protective nature of stigma-reduction and peer-support interventions, our findings likely underestimate the prevalence of symptoms of CMDs among MSM in Ghana. The sample size is small, which limits our ability to assess differences among sub-groups of MSM. While we were fortunate to be able to leverage the close-out of the RCT to measure the prevalence of mental health concerns, we are limited in our ability to investigate factors associated with poor mental health. However, the data presented provides useful insights into the magnitude of mental health concerns among MSM both with and without HIV.

## Conclusion

This descriptive short report presents valuable mental health prevalence data among a particularly vulnerable population, thus helping to fill a crucial gap in the literature, particularly in West Africa. Further research is urgently needed to understand risk and protective factors for CMDs among MSM in Ghana and elsewhere in sub-Saharan Africa. These findings also signal the need for investments in psychosocial support for MSM beyond HIV prevention and treatment as well as in expanding access to services for mental health promotion and treatment. Recognizing the reinforcing syndemics of HIV and mental health problems that hamper the wellbeing of MSM, culturally and contextually appropriate interventions that integrate in HIV and mental health care for MSM will be critical to addressing their healthcare needs [1].

## Supporting information

S1 Appendix Assessment of missingness and impact of imputation. (DOCX)

S1 Checklist Inclusivity checklist. (DOCX)

## References

[1] Operario D, Sun S, Bermudez AN, Masa R, Shangani S, van der Elst E. Integrating HIV and mental health interventions to address a global syndemic among men who have sex with men. The Lancet HIV. 2022. 10.1016/S2352-3018(22)00076-5 PMC7613577 35750058

[2] Singer M, Bulled N, Ostrach B, Mendenhall E. Syndemics and the biosocial conception of health. Lancet. 2017;389(10072):941–50. doi: 10.1016/S0140-6736(17)30003-X 28271845

[3] Beyrer C, Sullivan P, Sanchez J, Baral SD, Collins C, Wirtz AL, et al. The increase in global HIV epidemics in MSM. AIDS. 2013;27(17):2665–78. doi: 10.1097/01.aids.0000432449.30239.fe 23842129

[4] Liu M, Cai X, Hao G, Li W, Chen Q, Chen Y, et al. Prevalence of Intimate Partner Violence Among Men Who Have Sex With Men: An Updated Systematic Review and Meta-Analysis. Sex Med. 2021;9(6):100433. doi: 10.1016/j.esxm.2021.100433 34571326 PMC8766270

[5] Ogunbajo A, Iwuagwu S, Williams R, Biello KB, Kahler CW, Sandfort TGM, et al. Experiences of minority stress among gay, bisexual, and other men who have sex with men (GBMSM) in Nigeria, Africa: The intersection of mental health, substance use, and HIV sexual risk behavior. Glob Public Health. 2021;16(11):1696–710. doi: 10.1080/17441692.2020.1834598 33108249 PMC8076332

[6] Harper GW, Crawford J, Lewis K, Mwochi CR, Johnson G, Okoth C. Mental health challenges and needs among sexual and gender minority people in Western Kenya. International Journal of Environmental Research and Public Health. 2021;18(3):1311. 33535647 10.3390/ijerph18031311 PMC7908383

[7] Jauregui JC, Mwochi CR, Crawford J, Jadwin-Cakmak L, Okoth C, Onyango DP, et al. Experiences of Violence and Mental Health Concerns Among Sexual and Gender Minority Adults in Western Kenya. LGBT Health. 2021;8(7):494–501. doi: 10.1089/lgbt.2020.0495 34463158

[8] Sandfort TGM, Bos H, Reddy V. Gender Expression and Mental Health in Black South African Men Who Have Sex with Men: Further Explorations of Unexpected Findings. Arch Sex Behav. 2018;47(8):2481–90. doi: 10.1007/s10508-018-1168-9 29464453 PMC6102085

[9] Abubakari GM, Dada D, Nur J, Turner D, Otchere A, Tanis L, et al. Intersectional stigma and its impact on HIV prevention and care among MSM and WSW in sub-Saharan African countries: a protocol for a scoping review. BMJ Open. 2021;11(8):e047280. doi: 10.1136/bmjopen-2020-047280 34362801 PMC8351482

[10] Kushwaha S, Lalani Y, Maina G, Ogunbajo A, Wilton L, Agyarko-Poku T, et al. “But the moment they find out that you are MSM…”: a qualitative investigation of HIV prevention experiences among men who have sex with men (MSM) in Ghana’s health care system. BMC Public Health. 2017;17(1):770. doi: 10.1186/s12889-017-4799-1 28974257 PMC5627492

[11] Acts of ghana criminal code, 1960 (ACT 29). 1960.

[12] Nelson LE, Nyblade L, Torpey K, Logie CH, Qian H-Z, Manu A, et al. Multi-level intersectional stigma reduction intervention to increase HIV testing among men who have sex with men in Ghana: Protocol for a cluster randomized controlled trial. PLoS One. 2021;16(11):e0259324. doi: 10.1371/journal.pone.0259324 34843529 PMC8629287

[13] Wilton L, Herbst JH, Coury-Doniger P, Painter TM, English G, Alvarez ME, et al. Efficacy of an HIV/STI prevention intervention for black men who have sex with men: findings from the Many Men, Many Voices (3MV) project. AIDS Behav. 2009;13(3):532–44. doi: 10.1007/s10461-009-9529-y 19267264

[14] Dennis C-L. Peer support within a health care context: a concept analysis. Int J Nurs Stud. 2003;40(3):321–32. doi: 10.1016/s0020-7489(02)00092-5 12605954

[15] Kroenke K, Spitzer R, Williams J. The PHQ-9: validity of a brief depression severity measure. Gen Intern Med. 2001;16:606–13. 10.1046/j.1525-1497.2001.016009606.x PMC1495268 11556941

[16] Spitzer RL, Kroenke K, Williams JBW, Löwe B. A brief measure for assessing generalized anxiety disorder: the GAD-7. Arch Intern Med. 2006;166(10):1092–7. doi: 10.1001/archinte.166.10.1092 16717171

[17] Prins A, Bovin MJ, Smolenski DJ, Marx BP, Kimerling R, Jenkins-Guarnieri MA, et al. The Primary Care PTSD Screen for DSM-5 (PC-PTSD-5): Development and Evaluation Within a Veteran Primary Care Sample. J Gen Intern Med. 2016;31(10):1206–11. doi: 10.1007/s11606-016-3703-5 27170304 PMC5023594

[18] Institute for Health Metrics and Evaluation. GBD 2019 Cause and Risk. University of Washington. 2020. https://www.healthdata.org/research-analysis/health-risks-issues/mental-health

[19] Stoloff K, Joska JA, Feast D, De Swardt G, Hugo J, Struthers H. A description of common mental disorders in men who have sex with men (MSM) referred for assessment and intervention at an MSM clinic in Cape Town, South Africa. AIDS and Behavior. 2013;17:77–81. 10.1007/s10461-013-0430-3 23532397

[20] Ahaneku H, Ross MW, Nyoni JE, Selwyn B, Troisi C, Mbwambo J, et al. Depression and HIV risk among men who have sex with men in Tanzania. AIDS Care. 2016;28 Suppl 1(sup1):140–7. doi: 10.1080/09540121.2016.1146207 27002772 PMC4859320

[21] Stahlman S, Grosso A, Ketende S, Sweitzer S, Mothopeng T, Taruberekera N. Depression and social stigma among MSM in Lesotho: implications for HIV and sexually transmitted infection prevention. AIDS and Behavior. 2015;19:1460–9. 25969182 10.1007/s10461-015-1094-y PMC4858167

[22] Yu Y, Wang X, Wu Y, Weng W, Zhang M, Li J, et al. The benefits of psychosocial interventions for mental health in men who have sex with men living with HIV: a systematic review and meta-analysis. BMC Psychiatry. 2022;22(1):440. doi: 10.1186/s12888-022-04072-1 35768860 PMC9241196

[23] Mulqueeny DM, Nkabini SM, Pokiya MH. Mapping evidence of depression in HIV-seropositive MSM in sub-Saharan Africa: a scoping review protocol. Syst Rev. 2021;10(1):50. doi: 10.1186/s13643-021-01604-w 33546768 PMC7866456

[24] Xiao L, Qi H, Wang Y-Y, Wang D, Wilkinson M, Hall BJ, et al. The prevalence of depression in men who have sex with men (MSM) living with HIV: A meta-analysis of comparative and epidemiological studies. Gen Hosp Psychiatry. 2020;66:112–9. doi: 10.1016/j.genhosppsych.2020.04.001 32818791

[25] Ayano G, Tsegay L, Solomon M. Food insecurity and the risk of depression in people living with HIV/AIDS: a systematic review and meta-analysis. AIDS Res Ther. 2020;17(1):36. doi: 10.1186/s12981-020-00291-2 32571426 PMC7310141

[26] Zhao Y, Khoshnood K, Sheng Y. Social support as a mediator between mental health and stigma among newly HIV-positive men who have sex with men. Int J STD AIDS. 2024;35(6):477–86. doi: 10.1177/09564624241227653 38314565

[27] Smit PJ, Brady M, Carter M, Fernandes R, Lamore L, Meulbroek M, et al. HIV-related stigma within communities of gay men: a literature review. AIDS Care. 2012;24(4):405–12. doi: 10.1080/09540121.2011.613910 22117138 PMC3379736

[28] Doshi M, Macharia P, Mathenge J, Musyoki H, Amico KR, Battacharjee P. Beyond biomedical and comorbidity approaches: Exploring associations between affinity group membership, health and health seeking behaviour among MSM/MSW in Nairobi, Kenya. Global Public Health. 2020;15(7):968–84. 32172670 10.1080/17441692.2020.1739729

[29] Barthel D, Barkmann C, Ehrhardt S, Bindt C, International CDS Study Group. Psychometric properties of the 7-item Generalized Anxiety Disorder scale in antepartum women from Ghana and Côte d’Ivoire. J Affect Disord. 2014;169:203–11. doi: 10.1016/j.jad.2014.08.004 25212996

[30] Anum A, Adjorlolo S, Kugbey N. Depressive symptomatology in adolescents in Ghana: Examination of psychometric properties of the Patient Health Questionnaire-9. J Affect Disord. 2019;256:213–8. doi: 10.1016/j.jad.2019.06.007 31181377

[31] Barthel D, Barkmann C, Ehrhardt S, Schoppen S, Bindt C, International CDS Study Group. Screening for depression in pregnant women from Côte d׳Ivoire and Ghana: Psychometric properties of the Patient Health Questionnaire-9. J Affect Disord. 2015;187:232–40. doi: 10.1016/j.jad.2015.06.042 26343851

