# Supplementary material for: Symptoms of common mental disorders and suicidality among sexually diverse men who have sex with men in Ghana
Source: PLOS Ment Health. 2025 Jul 3;2(7):e0000331. doi: 10.1371/journal.pmen.0000331 (PMC12782570; doi:10.1371/journal.pmen.0000331)
Supplement: S1 Appendix — (DOCX) [file pmen.0000331.s001.docx]

**S1 Appendix:** **Assessment of Missingness and Impact of Imputation**

- *For depression, only 7 participants had missing data; 6 participants were missing 1 question and 1 participant was missing 2 questions. Treating scores to missing questions as “0”, of the 6 missing 1 question: 2 participants had a pre-imputation total score of 1, 1 participant had a pre-imputation total score of 5, and 1 participant had a pre-imputation total score of 11. These individuals would be correctly categorized as having no, mild and moderate depression, respectively regardless of their response to the missing question. For example, the maximum score to any PHQ-9 question is 3. Thus, an individual missing 1 question with a pre-imputation total score of 1 is 4 and would be correctly categorized regardless as to how that individual would have responded to the missing question.* ***As a result, imputation only could have impacted the categorization of 3 of the 7 participants with missing data.***
- *For PTSD, of the 168 participants who responded to the trauma gate question, only 4 participants had missing data, all missing 1 question. Treating scores to missing questions as “0”, of these 4 participants, 1 participant had a total pre-imputation score of 1, 2 participants had a total pre-imputation score of 3, and 1 participant had a total pre-imputation score of 4. Again, because the maximum score to any PTSD question is 1, the participants with a pre-imputation total score of 1 and 4 would be correctly categorized as not reporting PTSD and reporting PTSD regardless of their response to the missing question.* ***As a result, imputation only could have impacted the categorization of 2 of the 4 participants with missing data.***
- *For anxiety, we recognize that imputation could have had a larger impact on the prevalences reported given we imputed scores for 54 individuals (52 individuals missing responses to 1 question and 2 individuals missing responses to 1 question). To integrate the impact of this imputation, again we assess the pre-imputation scores (treating missing responses as a score of “0”). Of the 52 individuals missing response to 1 question, 4 individuals had a pre-imputation total score of 0, 4 individuals had a pre-imputation total score of 1, 7 individuals had a pre-imputation total score of 5, 9 individuals had a pre-imputation total score of 6, 3 individuals had a pre-imputation total score of 10, and 1 individual had a pre-imputation total score of 11 Again, because the maximum score to any GAD-7 question is 3, the participants with a pre-imputation total score of 0 or1, 5 or 6, and 10 or 11 would be correctly categorized a no, mild, and moderate anxiety regardless of their response to the missing question.* ***As a result, imputation only could have impacted the categorization of 26 of the 54 individuals with missing data.***

**Companion Table 2.2, Exploration of Impact of Imputation**

|  | **Post Imputation**  (Table 2 in Manuscript) | **Pre Imputation** (Treating Missing as "0") | **Complete Case** (Excluding participants with missing data*) | **Categorizable without Imputation**  (excluding only those with missing response(s) that could have impacted the categorization**) |
| --- | --- | --- | --- | --- |
|  | **Depression (n=186)** | **Depression (n=186)** | **Depression (n=179)** | **Depression (n=183)** |
| Mild (PHQ-9 5-9) | 59 (31.7%) | 59 (31.7%) | 57 (31.8%) | 58 (31.7%) |
| Moderate (PHQ-9 10-14) | 27 (14.5%) | 27 (14.5%) | 26 (14.5%) | 27 (14.8%) |
| Severe (PHQ-9 ≥ 15) | 11 (5.9%) | 11 (5.9%) | 11 (6.1%) | 11 (6%) |
|  | **Anxiety (n=186)** | **Anxiety (n=186)** | **Anxiety (n=132)** | **Anxiety (n=160)** |
| Mild (GAD-7 5-9) | 71 (38.2%) | 74 (39.8%) | 47 (35.6%) | 63 (39.4%) |
| Moderate (GAD-7 10-14) | 20 (10.7%) | 16 (8.6%) | 11 (8.3%) | 15 (9.4%) |
| Server (GAD-7 ≥ 15) | 3 (1.6%) | 2 (1.1%) | 2 (1.5%) | 2 (1.3%) |
|  | **PTSD (n=168)** | **PTSD (n=168)** | **PTSD (n=164)** | **PTSD (n=166)** |
| Experienced traumatic event | 105 (62.5%) | 105 (62.5%) | 101 (61.6%) | 103 (62%) |
| PTSD (PC-PTSD-5 ≥4) | 53 (31.5%) | 53 (31.5%) | 49 (29.9%) | 51 (30.7%) |

*Depression missing n=7; Anxiety missing n=54; PTSD missing n=4

**Depression missing n=3; Anxiety missing n=36; PTSD missing n=2 (see description above)
